# Validity of demirjian and nolla methods for dental age estimation for Northeastern Turkish children aged 5–16 years old

**DOI:** 10.4317/medoral.18034

**Published:** 2012-05-01

**Authors:** Bilge Nur, Adem Kusgoz, Mehmet Bayram, Mevlut Celikoglu, Metin Nur, Saadettin Kayipmaz, Sina Yildirim

**Affiliations:** 1 Research Assistant, DDS, Department of Pedodontics, Faculty of Dentistry, Karadeniz Technical University, Trabzon, Turkey; 2 Assistant Professor, DDS, PhD, Department of Pedodontics, Faculty of Dentistry, Karadeniz Technical University, Trabzon, Turkey; 3 Assistant Professor, DDS, PhD, Department of Orthodontics, Faculty of Dentistry, Karadeniz Technical University, Trabzon, Turkey; 4 Assistant Professor, DDS, PhD, Department of Oral Diagnosis and Radiology, Faculty of Dentistry, Karadeniz Technical University, Trabzon, Turkey; 5 Research Assistant, DDS, Department of Orthodontics,, Faculty of Dentistry, Karadeniz Technical University, Trabzon, Turkey

## Abstract

Objective: To evaluate the applicability of Demirjian and Nolla methods for northeastern Turkish population. Material and Method: A retrospective study was performed on panoramic radiographs of 673 subjects aged 5–15.9 years. The mean dental age (DA) according to the Demirjian and Nolla methods were compared to the mean chronological age (CA). Results: The mean CA of the study sample was 10.37±2.90 and 10.03±2.81 years for females and males, respectively. Using the Demirjian method, the mean estimated DA was 11.26±3.02 years for females and 10.87±2.96 years for males. For Nolla method, the mean estimated DA was 9.80±3.41 and 9.53±3.14 years for females and males, respectively. The mean differences between the CA and DA according to the Demirjian and Nolla methods were 0.86 and -0.54 years for total study sample. Conclusion: Nolla method was found to be a more accurate method for estimating DA in northeastern Turkish population.

** Key words:**Dental age, demirjian method, nolla method, chronological age.

## Introduction

Chronological age (CA) is important in most societies for school attendance, employment, social benefits and marriage (1). However, there are many instances where CA might not be known due to the undocumented or missing birth data. One of the most accurate, reliable and fast method of age determination especially in the growing children is the dental method of age estimation (2). Sexual maturation characteristics, height, weight, and skeletal development have also been used to identify stages of growth. Of them, it was shown that the skeletal development was the most reliable method. The method most widely used for skeletal development determination is the reference atlas Greulich and Pyle, since it has the advantages of simplicity and availability of multiple ossification centers for the evaluation of maturity (3).

On the other hand, the importance of dental age (DA) has been emphasized in forensic dentistry and archeology (2,4). It can aid to the age identification of a deceased child. They have also been proven valuable when birth data are lacking or doubted in the management of immigration to help to determine physiologic age (4). Besides, in orthodontics and pediatric dentistry, more attention is usually paid to the patient’s DA rather than to their CA that both parameters do not always coincide (5). Sukhia et al. (6) noticed that orthodontic treatment might be started at a later stage in patients with delayed dental maturity, thus leading to shorter treatment duration and a more stable result.

DA may be assessed either by tooth eruption dates or by the progress of tooth calcification. Dental eruption is influenced by various factors such as crowding, extractions, ankylosis, ectopic positions, and persistence of primary teeth (7). In addition, tooth eruption dates cannot be applied between the ages of 3 and 6 years, or after the age of 13 years (8). Therefore, tooth calcification is thought to be a more reliable criterion for determining the DA.

Several methods (9-12) have been used to determine the DA according to the degree of the calcification observed in radiographic examinations in permanent teeth. Many authors (1,2,4,6,13-18) have tested the applicability of the DA assessment methods in their populations and most of them (2,6,13-16,18) have reported significant differences among the study samples and those methods. Willems et al. (1) tested the validity of Demirjian’s method on Belgian Caucasian population and observed consistent overestimation of the dental age in both sexes. They presented new tables for each sex with age score directly expressed in years. Chaillet et al. (19) conducted a research with 9577 dental panoramic radiographs of eight different ethnicities and implemented new international dental developmental weighted scores and curves for children with unknown ethnic origin. Cruz-Landeira et al. (20) tested the applicability of Demirjian’s and Chaillet’s method for Spanish and Venezuelan populations. They stated that the same method could produce a different result which means applicability of each method varies with a population used.

The aim of this study was to evaluate the applicability of Demirjian and Nolla methods for northeastern Turkish population, since little is known about those methods’ applicability for this population.

## Material and methods

The present study was a retrospective study conducted on panoramic radiographs of 673 subjects. The sample constituted of 342 males and 331 females with a mean CA of 10.03±2.81 years for males and 10.37±2.90 years for females. Panoramic radiographs from the subjects attending to the Faculty of Dentistry of the Karadeniz Technical University (Trabzon, Turkey) were included in the study. Northeastern Turkish subjects aged 5–15.9 years with no prior orthodontic treatment history and good quality of panoramic radiographs were included. Distribution of the subjects by gender and age is shown in ( Table 1). Subjects with systemic diseases affecting the growth and development of the teeth and tooth age-nesis other than third molars were excluded.

**Table 1:**
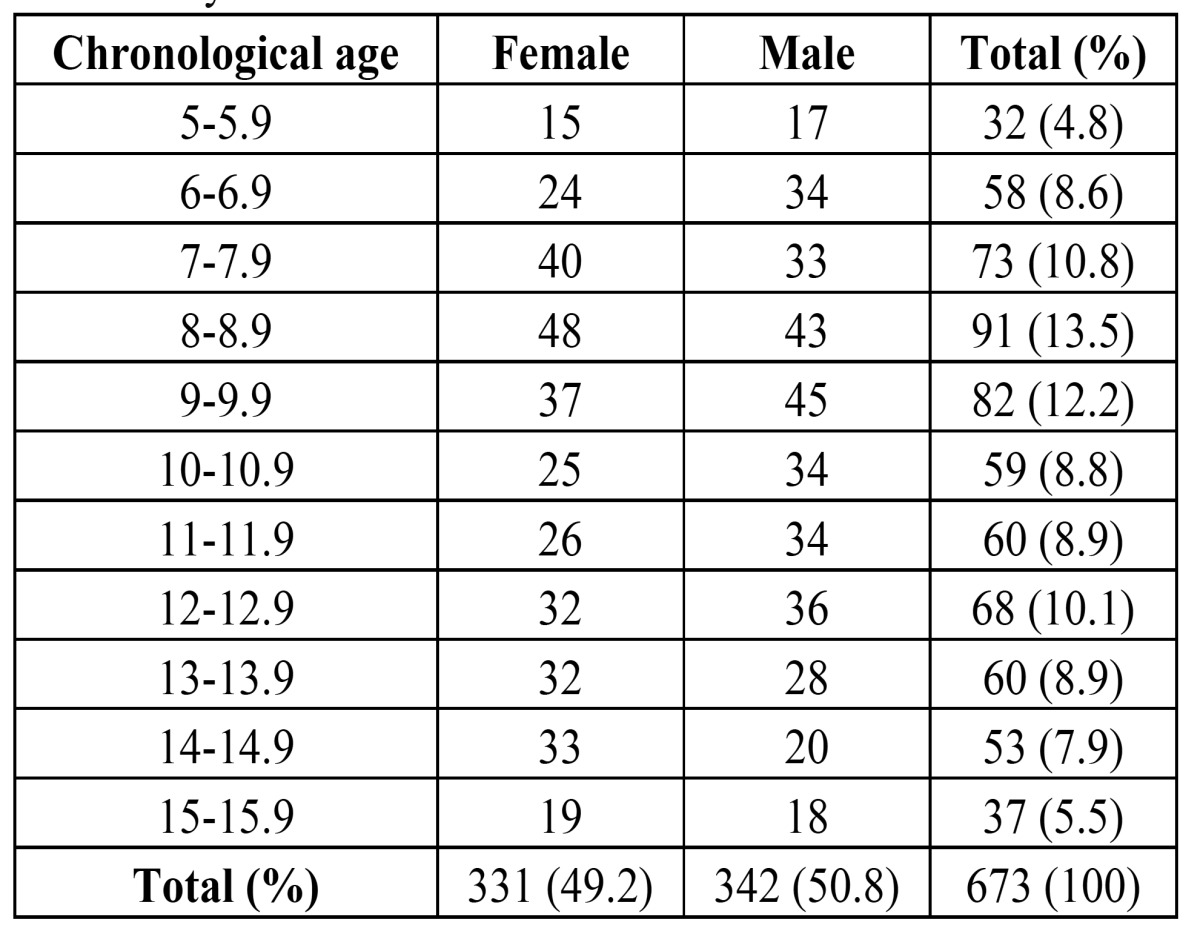
Age and gender distribution of the subjects included in the study.

All assessments were performed by one investigator in a darkened room with a radiographic illuminator to ensure contrast enhancement of the tooth images. In order to avoid the examiner bias at the time of collecting data, CA was first recorded on a data collection sheet and the DA scores were tabulated later on a separate sheet.

Chronologic Age

CA was calculated by subtracting the date of the birth from the date of the panoramic radiograph after having converted both to a decimal age.

Dental Age (Demirjian method)

The development of each left permanent mandibular tooth, except the third molar, was rated on an 8-stage scale from A to H, and the criteria for the stages were given for each tooth separately. Each stage of the seven teeth was allocated a score, and the sum of the scores gave an evaluation of the subject’s dental maturity, measured on a scale from 0 to 100. The dental maturity score of each subject was then converted to DA by using standard tables and/or percentile curves which were given for each gender, separately.

Dental Age (Nolla method)

Each left permanent mandibular tooth was assessed and assigned a stage of between 1 (no sign of calcification) and 10 (apical end completed). If the tooth was between stages an appropriate fraction (0.2, 0.5 or 0.7) was added as recommended by Nolla. The sum of scores was compared to the average sum for males or females and DA calculated.

Statistical Analyses

All descriptive and comparative statistical analyses were performed using the SPSS software package (Statistical Package for Social Sciences, version 11.5, SPSS Inc., Chicago, IL, USA). Kolmogorov-Smirnov test was performed to test the normality of the data. Since the results of the Kolmogorov-Smirnov test showed non-normal distribution, non parametric tests were performed. The DA and CA were compared using the Wilcoxon Signed Rank test. Besides, Spearman correlation coefficients for males, females, and total study sample were performed for both methods. To assess reproducibility, 60 randomly selected radiographs were reexamined four weeks after the initial examination by the same investigator. Examination of results using the Wilcoxon test showed no statistically significant differences between the two readings, indicating diagnostic reproducibility. A P value less than 0.05 was considered to be significant.

## Results

Differences between the mean CA and estimated DA using the Demirjian method for different age groups and total sample for males and females are presented in ( Table 2). Both genders were overestimated in dental maturity when compared with the reference samples. The mean difference between the CA and DA was 0.89±1.15 and 0.84±1.36 years for females and males, respectively. The mean difference between the CA and DA ranged from 0.15±1.13 to 1.24±0.57 years in females. These differences in females between the CA and DA were statistically significant in total (p<0.01) and in all groups (p<0.05) except for group of 15–15.9 years (p>0.05). The mean difference between the CA and DA ranged from 0.27±0.96 to 1.60±0.55 years in males. These differences in males between the CA and DA were statistically significant in total (p<0.01) and in all groups (p<0.05) except for groups of 13–13.9 and 14-14.9 years (p>0.05). The mean difference between CA and DA was 0.86±1.26 years in total (p<0.01).

**Table 2:**
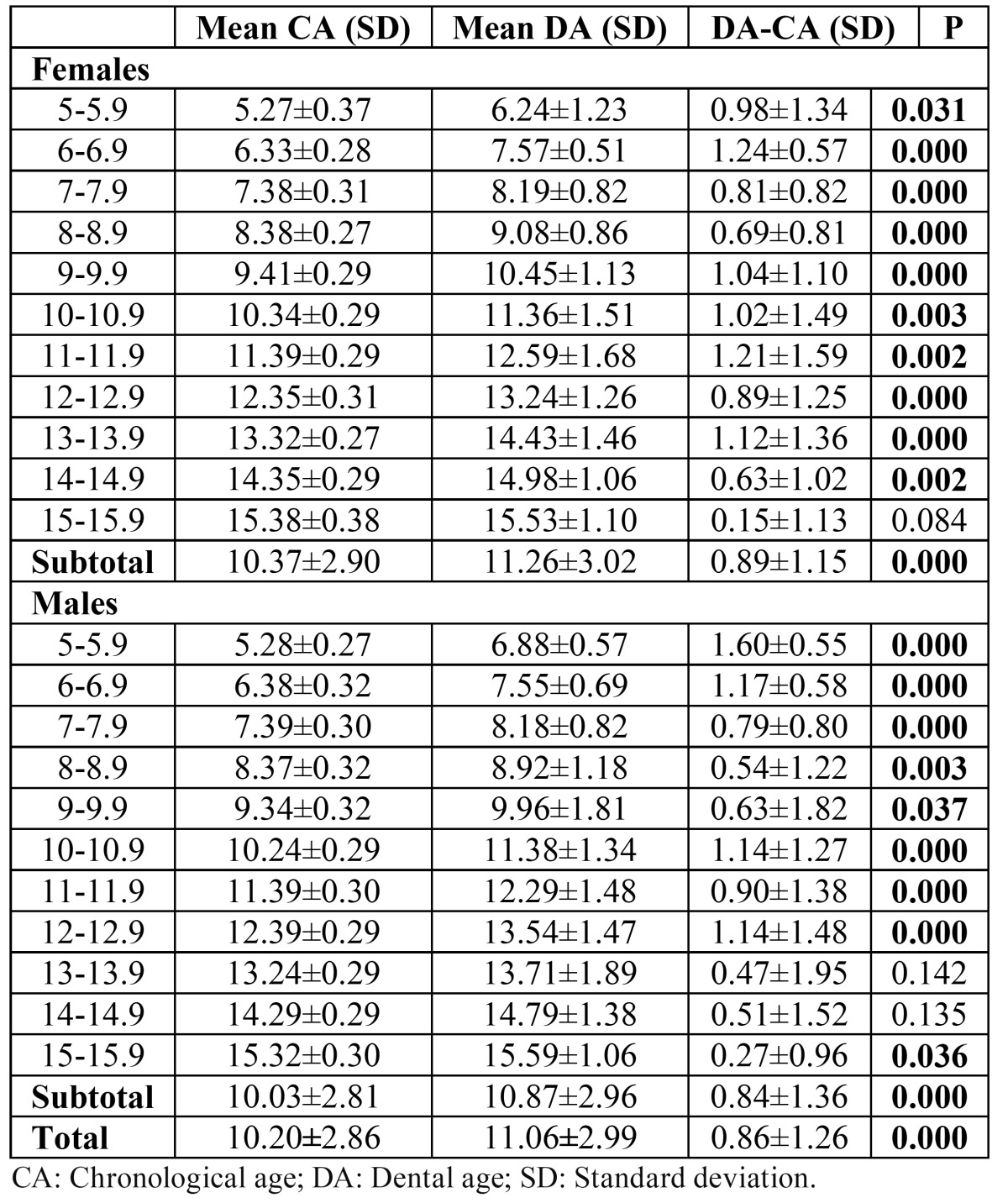
Differences between CA and DA determined by Demirjian’s method.

Differences between the mean CA and estimated DA according to the Nolla method for different age groups and total sample for males and females are presented in ( Table 3). Both genders were underestimated in dental maturity as compared to the reference samples. The mean difference between the CA and DA was -0.57±1.43 and -0.50±1.38 years for females and males, retrospectively. The mean difference ranged from -0.01±2.17 to -0.93±0.65 years for females and from -0.01±0.65 to -0.94±0.89 years for males. The differences between the CA and DA were statistically significant in total (p<0.01) and in 7-10.9 and 12-12.9 year age groups (p<0.05) for females and in total (p<0.01) and in 6-6.9 (p<0.05) and 8-11.9 (p<0.01) year age groups for males. The mean difference between CA and DA was -0.54±1.40 years in total (p<0.01).

**Table 3:**
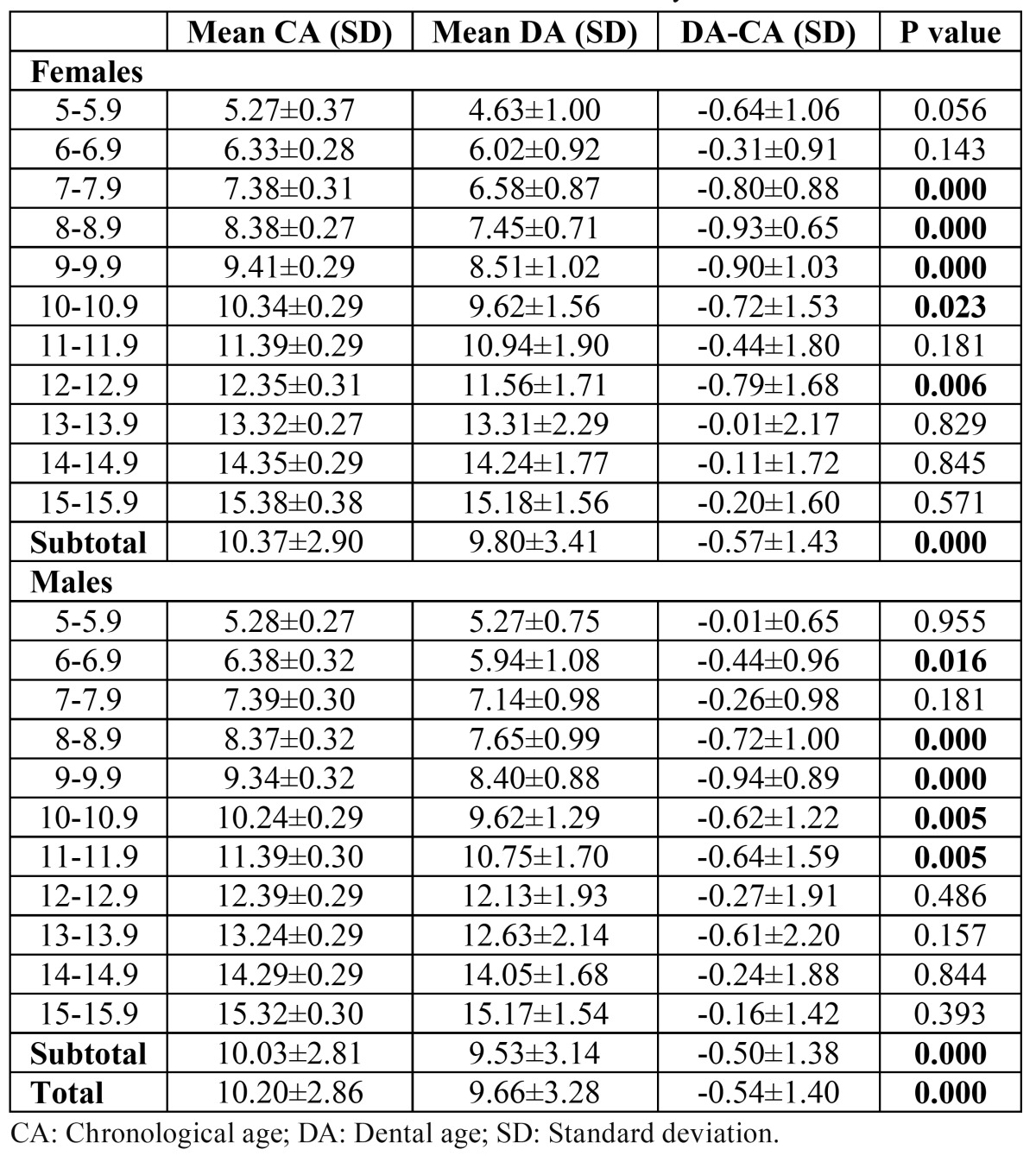
Differences between CA and DA determined by Nolla’s method.

**Figure 1:**
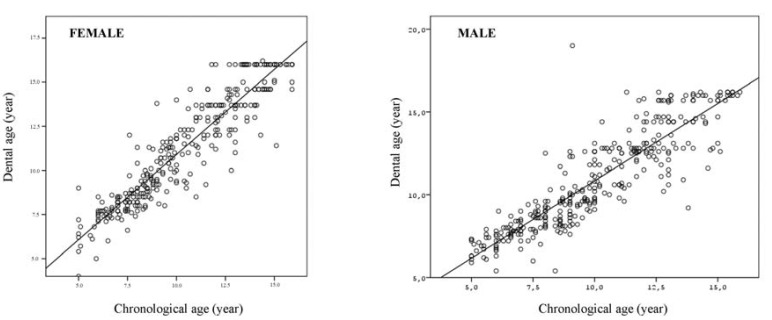
The scatter plot dental age according to the Demirjian method versus chronological age for males and females.

**Figure 2:**
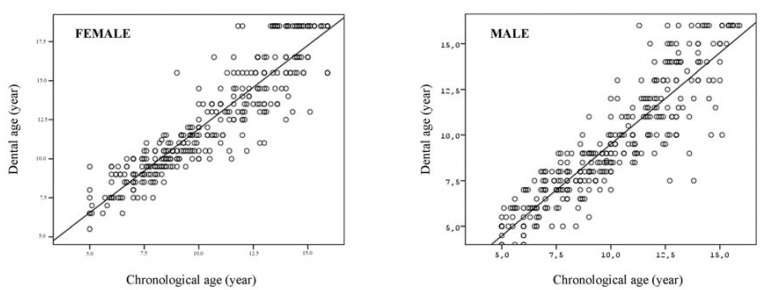
The scatter plot dental age according to the Nolla method versus chronological age for males and females.

The results of the Spearman correlation coefficients performed for total male and female samples according to the both methods are shown in ( Table 4). Results showed a strong linear correlation between CA and DA for both Demirjian method (for females and males; r2=0.931, r2=0.913, respectively) and Nolla method (for females and males; r2=0.928, r2=0.914, respectively). (Figs. 1 and 2) show the scatter plots of DA versus CA for females and males according to Demirjian and Nolla methods, respectively.

**Table 4:**
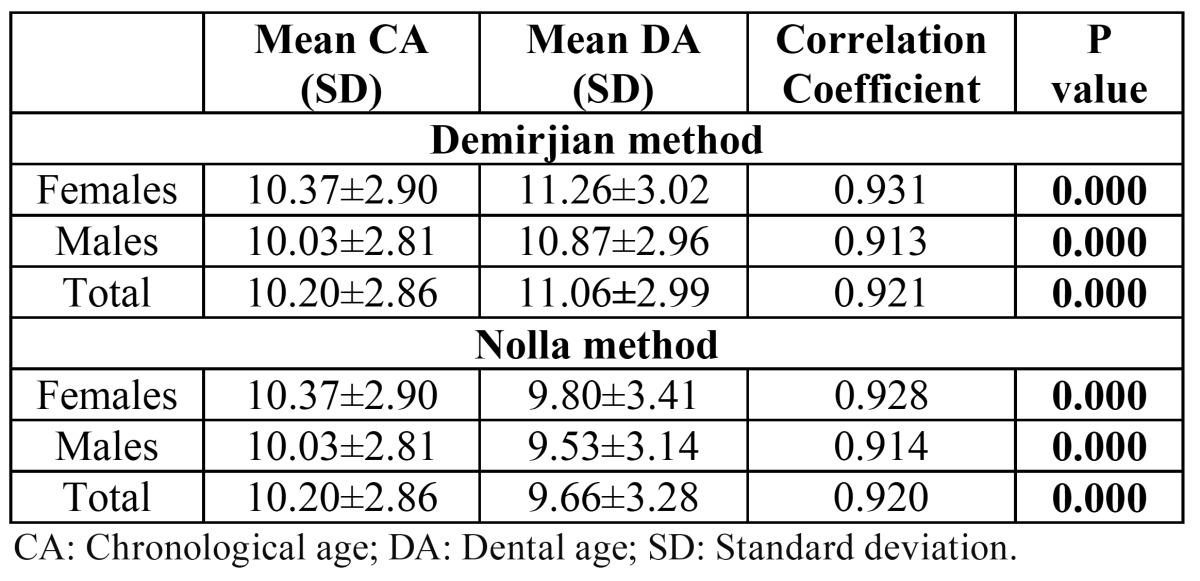
Results of the Spearman correlation coefficients performed for total male and female samples according to the both method.

## Discussion

DA assessments involve the use of radiographs and different types of radiographs have been used to investigate dental development. Panoramic radiographs have been adopted by most authors due to their accessibility and the possibility to visualize all teeth (2). Panoramic radiographs are also considered to be the best tool for age estimation in children, since intraoral radiographs are difficult to obtain without image distortion as distortion can lead to inaccuracy of findings (21). Thus, panoramic radiographs were used in this study to determine estimated DAs.

There have been several different methods to determine DA. Among all methods used to assess DA, the methods of Demirjian et al. (11) and Nolla are commonly used in teaching and clinical practice (4,21,22), hence these two methods were the obvious methods for the present study.

The method of Demirjian et al. (11) is one of the simplest, most practical, and widely employed methods to predict age and maturation (23,24). It is to determine the beginning of mineralization up to the end of root formation. The authors (11) looked at eight mineralization stages for premolars and molars (A–H) and six steps (C–H) for incisors and canines. However, it was based on a large sample of 1446 males and 1482 females of French-Canadian origin. They reported then that the possibility that the standards they obtained may not be valid in other populations and that perhaps adaptations should be made for other samples. Thus, numerous studies (1,2,6,7,13-15,18,25,26) have been conducted to determine the applicability of Demirjian’s method in a particular population. In the present study, both genders were overestimated in dental maturity for all age groups studied with a mean difference of 0.89 years in females and 0.84 years in males. Results from other studies that used the Demirjian method showed an ave-rage overestimation in dental age ranging from 0.02 to 3.04 years (13-15,18,21,25,27-29). This wide range might be due to the ethnic differences, climate, nutrition, socio-economic level, urbanization age structure of the study samples, sample size, statistical methods (4,14). In agreement with our findings, eastern Turkish children (18) were found to be dentally advanced and the differences were statistically significant in all age groups for both genders. Tunc and Koyuturk (15) evaluated the applicability of this method for DA estimation in northern Turkish children aged 4-12 years and found that the mean difference between DA and CA for males and females varied from 0.36 to 1.43 years and from 0.50 to 1.44 years, respectively. In this study, the mean difference between DA and CA for males and females was found to be varying from 0.27 to 1.60 years and from 0.15 to 1.24 years, respectively. In addition, the difference between DA and Ca was over 1 year for the ages 5-6.9, 9-11.9, 13-13.9 year old females and 5-6.9, 9-11.9, 13-13.9 years for males. However, the authors (15) noticed that it was over 1 year for the ages 5-6.9, 12-12.9 for females and 5-6.9 years for males. The difference between our findings and the authors’ findings might be due to the geographical differences and age range of the study sample.

Nolla method evaluates 10 mineralization stages of each tooth, two more degrees of mineralization of the crown than the more frequently used method according to Demirjian et al. (10,30). Maber et al. (4) compared the Nolla, Haavikko, Demirjian and Willems methods on a sample of children of Bangladeshi and British Caucasian ethnic origins. They found that Nolla method under-estimated age by -0.87 years for males and -1.18 years for females; also significantly different from chronological age (P < 0.01). Search of published reports showed that only one study (5) on dental age estimation according to the Nolla method among Turkish children has been reported. Miloglu et al. (5) noticed that the accuracy of the Nolla method was suitable for eastern Turkish males. They found an underestimation of a rate by 0.5 and 0.2 years for females and males, respectively. However, the mean difference between the CA and DA according to the Nolla method, in this study, was -0.57 and -0.50 years for females and males, respectively. The difference between our findings and their findings might be due to the differences between geographical areas as stated by several investigators (5,15).
